# Managing Benign and Malignant Oral Lesions with Carbon Dioxide Laser: Indications, Techniques, and Outcomes for Outpatient Surgery

**DOI:** 10.1055/s-0039-1694735

**Published:** 2019-08-05

**Authors:** Alberto Maria Saibene, Cecilia Rosso, Paolo Castellarin, Federica Vultaggio, Carlotta Pipolo, Alberto Maccari, Daris Ferrari, Silvio Abati, Giovanni Felisati

**Affiliations:** 1 Department of Otolaryngology, San Paolo Hospital, University of Milan, Milan, Italy; 2 Department of Biomedical, Surgical and Dental Sciences, Odontostomatology Unit, ASST Santi Paolo e Carlo, Università degli Studi di Milano, Milan, Italy; 3 Department of Oncology, San Paolo Hospital, University of Milan, Milan, Italy; 4 Department of Dentistry, Unit of Oral Pathology, IRCCS San Raffaele University Hospital, Vita-Salute San Raffaele University, Milan, Italy

**Keywords:** laser surgery, oral lesions, CO _2_, leukoplakia, benign oral lesions, outpatient procedures

## Abstract

**Purpose** Because of its affinity for water-based tissues, carbon dioxide (CO _2_ ) laser has become an instrument of choice for treating oral mucosa conditions, ranging from inflammatory to malignant lesions. The aim of this work is to systematically evaluate the outcomes of laser surgery over a wide range of lesions, while providing a solid and reproducible protocol for CO _2_ laser surgery in the outpatient management of oral lesion.

**Methods** Seventy-eight patients underwent 92 laser outpatient procedures for treatment of a wide range of benign and malignant lesions. We performed 60 removals, 11 exeretic biopsies, 15 vaporizations, and 3 vaporization/removal combined. We analyzed laser parameters applied for each technique and provided a systematic evaluation of surgical results.

**Results** No problems occurred intraoperatively in any of the patients. Five patients complained marginal pain, while 3 patients had postsurgery bleeding. All treatments were successful, with the notable exception of 3 relapsing verrucous proliferative leukoplakias and an infiltrating squamous cell carcinoma of the tongue requiring radicalization. We did not record any adverse reactions to drugs or lesions due to laser action. Concordance between clinical diagnosis and pathology results was at 94.8%.

**Conclusions** Our data indicate that CO _2_ laser is a solid choice for outpatient treatment of oral lesions. This technique grants painless and almost bloodless treatment, with negligible recurrence rates. Providing a solid reference for laser settings and operative techniques could provide a foundation for further exploring this tool while offering the basis for a positive comparison between different surgical techniques and options.

Carbon dioxide (CO _2_ ) laser was the first type of laser introduced in medical treatments. Producing beams of light at a 10,600-nm wavelength, which are absorbed by water in the tissue, it causes tissue hemostasis allowing excision of a large variety of soft tissue lesions, as well as many other clinical procedures.

It was invented in 1963 by Patel, then Ben-Bassat was the first to describe its use for intraoral treatment in 1984. Since then, many studies have endorsed the advantages and effectiveness of CO _2_ laser as treatment for oral (OLs), maxillofacial, and cervical lesions. 1

There are plenty of advantages of laser over cold steel surgical procedures such as minimal postoperative swelling and scarring. It also reduces bacterial contamination through sterilization of the surgical site. Compared with other laser types and to monopolar and bipolar cautery, it allows a reduced mechanical and thermal damage with minimal postoperative pain. It is becoming an establishment also for its anti-inflammatory, biostimulant, and regenerative effects, permitting excellent healing and low morbidity. 2 3 CO _2_ laser has been compared with other nonsurgical methods like diode laser and cryotherapy: CO _2_ and diode lasers have significantly better outcomes than cryosurgery in the management of oral leukoplakia in terms of pain, swelling, and slough formation. 4

Because of its affinity for water-based tissues, the CO _2_ laser has become a favorite instrument of oral surgeons for treatment of pathologic conditions of the oral mucosa. It has been recommended to treat benign OLs, such as fibromas, vascular anomalies, mucoceles, ranulae, gingival hyperplasias with different causes (idiopathic or due to side effects of medications), aphthous ulcers, mucosal frenula, or tongue ties (ankyloglossia), as well as premalignant lesions such as oral leukoplakia, erythroplakia, papillomas, and lichen planus. 4 5 6 7 8 Some reports on the use of the CO _2_ laser also support the possibility of treating malignant oral diseases in early stages with excisional biopsies, as well as mucosal melanomas. 2 9

In our institution, CO _2_ laser is used for outpatient treatment of a wide range of conditions: among benign lesions we adopt this procedure for treating traumatic pseudofibromas, scars, ulcers (traumatic or as a residual of surgical removal), fibromas, and mucoceles. Other common practice is the excision of potentially malignant neoformations like leukoplakias, pyogenic granulomas, papillomas, and actinic cheilitis. We also manage in situ and minimally invasive malignancies.

In the literature, most of the studies describe the utilization of CO _2_ laser for a single indication, over all for leukoplakia, but we lack papers methodologically exploring all possible utilizations for soft oral tissues surgery and the procedures to adopt in each singular occasion, especially with relevant caseloads. 3 5 6 7 This is peculiarly important if we take into account that modern CO _2_ lasers usually are equipped with software and beam scanners that allow not only cutting, but also vaporization, which has a traditional role in aesthetic skin treatments. Furthermore, current literature on the subject usually fails to provide methodological instructions on these treatments, with sparse information on scanning programs and laser power, thus making comparison between different techniques virtually impossible and raising the learning curve for untrained surgeons. Therefore, the aim of this work is to analyze a wide case series of outpatient treated at our institution to summarize current applications of these modern tools, while proposing valid CO _2_ settings and procedural instructions granting efficient OL treatment with marginal complications.

## Materials and Methods

The study has been designed as a retrospective review. Due to its retrospective design, it was granted exemption from the Institutional Review Board of ASST Santi Paolo e Carlo, Milan, Italy. We reviewed charts from patients attending our oropharyngeal pathology outpatient clinic to identify patients who underwent laser surgery for oral cavity conditions from June 2014 to May 2018. Inclusion criteria required outpatients diagnosed with benign or potentially malignant oral cavity lesions, CO _2_ laser outpatient treatment, and a minimum age of 14 years old to ensure an acceptable compliance. Patients with a follow-up period shorter than 30 days were excluded from the study. Seventy-eight consecutive patients were therefore identified. Patients' demographics are shown in Table 1 . They underwent 92 laser procedures, either lesion vaporization or removal; some patients received more treatments due to disease persistence, recurrences, or new onset lesions. The procedures are detailed in Table 2 .

**Table 1:** Analysis of patients' demographics: age average, standard deviation, and range are provided

Patients demographics	Specifics	Values
Age		
	Mean	56.7
	Standard deviation	15.55
	Range	14–83
Sex		
	Male	41
	Female	37

**Table 2:** Summary of surgical procedures considered in this study

Procedures and clinical diagnosis	*N*	%
Traumatic pseudofibroma removal	24	26.08
Papilloma removal	15	16.30
Leukoplakia removal	15	16.30
Verrucous proliferative leukoplakia vaporization	14	15.21
Leukoplakia exeretic biopsy	9	9.78
Granuloma removal	4	4.34
Ulcer removal	3	3.26
Scar vaporization	2	2.17
Lichen planus vaporization	2	2.17
Minor salivary glands mucocele removal	1	1.08
Leukoerythroplakia exeretic biopsy	1	1.08
Ulcer vaporization	1	1.08
Venous malformation removal	1	1.08
Total	92	100.00

Note: The condition treated is taken into account.

**Fig. 1:**
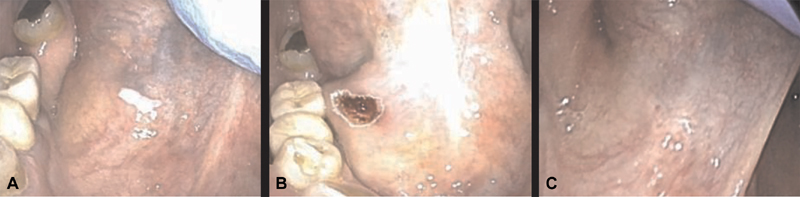
The picture shows a leukoplakia removal at the floor of mouth by carbon dioxide (CO _2_ ) laser. ( **A** ) Lesion before removal. ( **B** ) Lesion just after removal. ( **C** ) Lesion 3 weeks after removal.

**Fig. 2:**
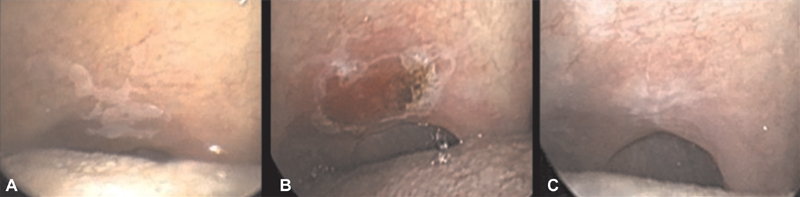
The picture shows a leukoplakia vaporization on soft palate by carbon dioxide (CO _2_ ) laser. ( **A** ) Lesion before vaporization. ( **B** ) Lesion just after vaporization. ( **C** ) Lesion 3 weeks after vaporization.

All patients were treated with a fractional CO _2_ laser (Lumenis Acupulse 30 ST, Lumenis Ltd, Yokneam, Israel) with a 125-mm handheld hand piece. Before any session of treatment, we registered a video of the lesion to document presurgical status of the pathology; an informed consent was obtained from the patient or his/her caregivers (for patients aged 14–18 years) prior to the treatments. After that, every procedure started with a local anesthesia with either Carbocaine alone or Carbocaine with adrenaline (the former was used in patients with already diagnosed heart conditions). Laser settings vary from removal to vaporization procedures; the former is performed through a 4-W continuous beam with superpulsed emission, with a 0.2 × 1 mm size, while the latter is performed through a 6-W superpulsed beam, with repeated emissions, with 0.49 seconds of range time between two consecutive pulses, a square or round beam shape, with 3 to 5 mm width, and 1 mm of depth (see Table 3 ). Our group performed, on 78 patients, 92 procedures: 71 cuttings, among which 63 complete removals and 11 incisional biopsies, and 18 vaporizations (see Table 4 ). Removals and biopsies were sent for surgical pathology examination, while vaporizations, which do not allow for surgical pathology examination, were performed only on patients with a confirmed diagnosis of verrucous proliferative leukoplakia or hyperkeratotic lichen planus oralis via prior punch biopsy. Leukoplakias were treated with laser excision whenever we deemed a surgical pathology examination useful for diagnosis ( Fig. 1 ), while we chose to vaporize lesions whose histology had been already confirmed with incisional biopsies no longer than 1 month before ( Fig. 2 ).

**Table 3:** Classification of technique typologies

Type of procedure	*N*	%
Removal	63	68.54
Vaporization	18	20.22
Biopsy	11	11.24

**Table 4:** Areas of treatment

Site of treatment		*N* of sessions	%
Tongue		27	30.00
	Papilloma	2	
	Cistoadenoma	1	
	Fibroma	9	
	Leukoplakia	13	
	Ulcer	4	
	Granuloma	3	
Cheek		17	18.89
	Leukoplakia	9	
	Fibroma	6	
	Granuloma	1	
	Papilloma	1	
Hard palate		11	12.22
	Leukoplakia	5	
	Papilloma	4	
	Fibroma	2	
Gum		10	11.11
	Leukoplakia	5	
	Fibroma	2	
	Papilloma	1	
	Venous-arterious malformation	1	
	Granuloma	1	
Inferior lip		8	8.89
	Fibroma	3	
	Scar	2	
	Leukoplakia	1	
	Papilloma	1	
	Mucocele	1	
Soft palate		5	5.56
	Papilloma	3	
	Leukoplakia	2	
Retromolar trigone		3	3.33
	Leukoplakia	3	
Frenulum of tongue		3	3.33
	Papilloma	3	
Oral floor		3	3.33
	Leukoplakia	2	
	Papilloma	1	
Oral vestibule		2	2.22
	Leukoplakia	1	
	Venous-arterious malformation	1	
Superior lip		1	1.11
	Papilloma	1	
Total		90	100.00

Note: Several procedures developed on two or more zones.

All patients were prescribed to apply during the following 2 weeks two gels on the surgical site: a 0.5% chlorhexidine gel and amino acid and hyaluronate gel. All patients were prescribed to use acetaminophen 1 g every 6 hours in case of mild to moderate pain. We attended outpatients for a follow-up at 21 days after treatment with a recorded video of the outcomes. All patients were instructed to contact our clinic in case of bleeding, worsening swelling, numbness, or intense/uncontrolled pain. Visual Analogue Scale (VAS) of 100 mm in length was used to evaluate the intensity of pain. This scale was converted into a numerical value: from 0 (corresponding to no pain) to 10 (unbearable pain). Each patient marked the intensity of pain at 24 hours, 48 hours, and then at 7 days postoperatively. To record healing time, we gave patients instructions of recording how many days after surgery the lesion healed.

We also analyzed clinical diagnosis divided by sites, making an evaluation of prevalence of each lesion for area of treatment. Finally, recurrences localization was investigated.

## Results

No problems occurred intraoperatively in any of the patients, and surgery was quickly and safely performed. No procedure took longer than 10 minutes. We had a negligible amount of complications: four patients had postsurgery bleeding, only two of them required cautery by bipolar forceps and one had absorbable stitches. The team only registered three relapses on verrucous proliferative leukoplakia: a wide recurrence which required a removal in general anesthesia, and two relapses at 6 and 24 months which respectively have been managed with an additional vaporization and a new biopsy with vaporization. A histological result of infiltrating squamous cell carcinoma of the tongue needed a radicalization through a following excision in general anesthesia associated with neck dissection. We did not record any adverse reactions to drugs or lesions due to laser action. Furthermore, laser CO _2_ seems a painless surgery due to the fact that most of the patients recorded a low pain level, with a mean of 2.5 on VAS scale after 24 hours and standard deviation of 1.78, a value of 2 after 48 hours with a standard deviation of 1.33, and 0 points 7 days after surgery. Only 2 patients differed from those estimates with a higher level of discomfort respectively at 8 and 7 points on VAS scale at 24 hours and 6 and 5 at 48 hours. Two patients complained temporary numbness around the operated area, which regressed within 2 and 5 days, respectively.

Healing time varied in a range from 10 to 13 days, with a mean of 11.89 days and a standard deviation of 1.87. Only 2 patients required a period of regeneration of 20 days, while 3 patients reported complete repair within 7 days.

**Fig. 3:**
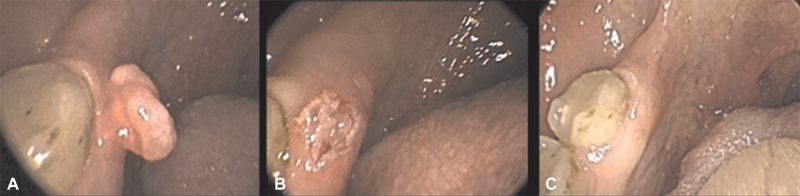
Fibroma removal on gingival surface by carbon dioxide (CO _2_ ) laser. ( **A** ) Lesion before removal. ( **B** ) Lesion just after removal. ( **C** ) Lesion 3 weeks after removal.

The procedure with the higher rate of application had been the exeresis of fibromas (26.08%) ( Fig. 3 ), followed by removal of papillomas (16.30%) and removal (16.30%) and vaporization (15.21%) of leukoplakias ( Table 2 ). Analysis revealed that the most treated area was the tongue (29.67%), followed by cheek mucosa (18.68%) and hard palate (12.09%) ( Table 5 ). We elaborated our data and discovered that most of leukoplakias (37.14%) have been shown in the tongue, 25.7% at the cheek mucosa. Gum and hard palate had a medium incidence of potentially malignant lesions: both at 14.28%; while lips, soft palate, oral floor, and other sites showed a nonrelevant incidence of leukoplakias.

**Table 5:** Summary of histological exams of the samples collected with biopsies and removals

Histological results	*N*	%
Traumatic fibroma	21	30.00
Papilloma	15	21.43
Hyperplasia	11	15.71
Granuloma	4	5.71
High grade dysplasia	3	4.29
Low grade dysplasia	3	4.29
Hyperkeratosis with orthokeratosis/parakeratosis	3	4.29
Ulcer	3	4.29
Infiltrating squamous cell carcinoma	1	1.43
In situ carcinoma	1	1.43
Actinic cheilitis	1	1.43
Acanthosis	1	1.43
Cistoadenoma	1	1.43
Venous malformation	1	1.43
Mucocele of minor salivary glands	1	1.43
Total	70	100.00

Considering only specimens sent to surgical pathology evaluation, we had a correlation of 94.8% between clinical diagnosis and anatomopathological results: 4 lesions proved to have different nature than we expected from clinical evaluation. Three patients with a clinical diagnosis of pseudofibroma were indeed diagnosed respectively with a cistoadenoma, a verrucous proliferative hyperplasia, and a hyperkeratosis, while a traumatic ulcer proved to be an infiltrating squamous cell carcinoma.

Histological results faithfully replicated clinical diagnosis, despite a little incongruence of percentage: fibromas represented 30% of the samples, papillomas 21.43%, and verrucous proliferative hyperplasias characterized 15.71% of the results ( Table 6 ).

**Table 6:** Setting of Lumenis CO _2_ laser parameters

Setting laser parameters	Exeresis – Biopsy	Vaporization
Wave	Continuous	Pulsed (0.49 s of interpulses pause)
Power	4 W	6 W
Length	1 mm	3–5 mm
Depth	n/a	1 mm
Beam shape	Spot	Round/square according to local anatomy
Scanner program	Cutting laser for general surgery	Feather touch/Silk touch for aesthetic surgery

## Discussion

Several studies discussed the competencies of laser technologies for OLs, as the development of this technique is moving toward a painless and bloodless oral surgery. 2 3 4 Our work sustains this thesis and confirms CO _2_ laser as a valid option for treatment of OLs as biopsy, vaporization, or removal.

Comparing to others' works shown in the literature, ours focused on an overall analysis of laser application on outpatients' oral surgery. Moreover, extending the review to different types of lesions among the benign ones, like granulomas, fibromas, or papillomas, and the potentially malignant ones, as leukoplakias, allows to provide a way of comparison not only of results but also of the specific details of laser techniques that are employed. Laser settings are indeed an almost neglected aspect into the specialized literature, which fails in providing specific information over this kind of procedure. Clarifying the parameters and methods of laser technique has the purpose of furnishing a basis for anyone who approaches for the first time this kind of surgery, but it is also a benchmark for comparison between wiser surgeons already familiar with this practice. Although our works lacks in terms of novel findings, our article covers a significant gap in the literature, providing a sort of “beginners' guide” for laser surgery, helping newcomers to this technique and providing a reference for treatment options in a solid patients series.

Many works also investigated bleeding resulting from CO _2_ laser procedures: Goodson et al calculated a 4.87% of bleeding as complication of laser CO _2_ treatment of cancers and precancerous OLs. 10 Our evaluation matched this data with a 5.12% of bleeding cases after procedure.

A study investigated recurrences of oral potentially malignant disorders over 773 laser procedures: 9% of them showed persistent disease, and most of them were verrucous leukoplakias. Unexpected oral squamous cell carcinoma was detected in 12%, while 12% evolved into malignancies. Another article registered 19.5% of recurrences in dysplastic lesions, and 10.4% of malignant progression in a mean of 6.4 years' follow-up. 9 11 A retrospective review of 65 laser-treated leukoplakias found 33.8% of relapses and 15.4% of progression rate along 15 months. 8

In our group, we identified 3 relapses over 89 procedures on benign and potentially malignant lesions; recurrences occurred in only leukoplakia formations, demonstrating a rate of 13.63% of potentially malignant lesions recurrence. Two of them were at the tongue (66.6%), and one was at hard palate (33.3%).

CO _2_ laser has been widely explored and compared with other surgery methods like conventional scalpel. A recent study found CO _2_ surgery as a better technique for intraoperative hemostasis and reduced scarring; there were not any significant differences in terms of postoperative pain and swelling. 12 Natekar et al evaluated differences between CO _2_ laser, diode laser, and cryosurgery, and found laser methods to be painless and without any swelling or thermal damage, while cryosurgery recorded 30% of scar formation, 40% of swelling, and a higher pain score. 4 However, there was a total absence of bleeding in cryosurgery group, while the CO _2_ and diode laser surgery groups showed clinically negligible bleeding tendencies. Another work confirmed the advantage of CO _2_ laser over cold knife procedure in terms of pain and swelling. 13 All of these procedures required a following hemostasis.

Several authors agree that hystological dysplasia’s degree isn’t a predictor of recurrence or malignant transformation for OLs. Therefore, in literature it’s still debated whether to routinely manage these lesions with a surgical approach or not. 14

It has to be noted also that our sample reports a very small percentage of unexpected incidence of squamous carcinomas compared with recent literature reports 15 (1.43% vs. 12%). This striking differences is most probably due to our choice of treating as outpatient cases only patients with none to negligible chances of malignancy or patients with extremely small lesions (as in the case of the only in situ carcinoma we treated in this series). Choosing to manage with CO _2_ laser all kind of lesions, irrespectively of their malignancy risk, might therefore determine a higher number of unexpected carcinomas.

Several authors also investigated outcomes in term of pain and healing time. López-Jornet et al found that pain and swelling reported by patients was greater with the cold knife than with the CO _2_ laser. 13 While Tambuwala et al found a nonstatistically significant difference between pain occurred by CO _2_ laser and scalpel; the former set at 2.2 points on VAS score 1 day after surgery, and 1.2 two days postoperatively. 12 Another work on pediatric laser treatments reported a score of 0 on VAS scale 1 day after treatment in 86.8% of treated children. 16 They also confirmed the literature's findings of approximately 2 weeks' healing time for this kind of procedure. Other researchers showed a longer time of recovery, stated at 3 weeks. 17 18 We sustained former thesis showing a mean of 2.5 on VAS scale after 24 hours, a value of 2 after 48 hours, and 0 points 7 days after surgery. The average of healing time was in a range from 10 to 13 days, further confirming the best literature data currently available on CO _2_ laser surgery, also once again on a wider range of lesions.

Another critical point of laser therapy is about the thermal damage that it might cause on peri-incisional margins during a biopsy, affecting the histopathological features and creating doubts about diagnosis. 19 Many studies focused on this topic: Rizoiu et al showed no differences in the histology among peri-incisional margins of samples excised by laser and by scalpel; Romeo et al demonstrated in two articles how, even if lasers caused slight alterations in the taken tissue margins, no one of Er:YAG, Nd:YAG, Er-Cr:YSGG, and diode laser compromised the histological evaluation. 19 20 21 Even biopsies of suspicious lesions should not worry about the thermal effects on peripheral margins: they do not affect the analysis of cellular infiltration in the adjacent tissues and permit to establish the real cancer size.

Our study reflects those finding, though it is built on a relatively limited case series and it is made on a retrospective review, which does not allow us to pursuit a long-term follow-up or a standardization of parameters' collection. Besides, the results could not have been compared with a control group of scalpel excision or with other surgical lasers.

We also investigated the effect of vaporization on oral premalignant lesions, as we performed 9 vaporizations on leukoplakias and lichen planus which did not show any relapse, 3 procedures of removal and vaporization in the same session, and 2 vaporized lesions which evinced a recurrence. The rate of relapse among vaporizations has been 13.3%. On the other hand, we performed 11 laser excisions over leukoplakias, where we had a relapse on only one case (9%). A study from Brouns et al assessed the annual recurrence rate among 35 leukoplakias' vaporizations at 8%, and a malignant transformation rate at 3%. 22 Another study evaluated the risk of early leukoplakia recurrence within 3 months following CO _2_ laser removal varying by clinical characteristics including lesion size, site, and accessibility of margins. It came out how poor accessibility of the lesion margins is a predictor for early recurrence. 23 Finally, Del Corso et al made a comparison between laser evaporations and laser excision for the treatment of oral leukoplakias: they reported no significant differences for recurrences; however, CO _2_ excision revealed statistically better results with the nonhomogeneous OLs and OLs with mild dysplasia. 24

## Conclusion

Despite the above described limitations, this work permitted to highlight strongly positive outcomes on outpatients' laser surgery for oral lesions treatment. Aside from the clinical results and the low level of complications that we observed, what mostly stands out is the flexibility of laser as a complete device for the management of all kinds of lesions, merging the properties of removal technique with those of the vaporization, even in the same surgery session.
